# Identification of Solid and Liquid Materials Using Acoustic Signals and Frequency-Graph Features

**DOI:** 10.3390/e25081170

**Published:** 2023-08-05

**Authors:** Jie Zhang, Kexin Zhou

**Affiliations:** 1 School of Computer Science & Technology, Xi’an University of Posts & Telecommunications, Xi’an 710121, China; zhou@stu.xupt.edu.cn; 2 School of Information Science and Technology, Northwest University, Xi’an 710127, China

**Keywords:** material identification, image features, acoustic signals, mutual information, ECOC learning method

## Abstract

Material identification is playing an increasingly important role in various sectors such as industry, petrochemical, mining, and in our daily lives. In recent years, material identification has been utilized for security checks, waste sorting, etc. However, current methods for identifying materials require direct contact with the target and specialized equipment that can be costly, bulky, and not easily portable. Past proposals for addressing this limitation relied on non-contact material identification methods, such as Wi-Fi-based and radar-based material identification methods, which can identify materials with high accuracy without physical contact; however, they are not easily integrated into portable devices. This paper introduces a novel non-contact material identification based on acoustic signals. Different from previous work, our design leverages the built-in microphone and speaker of smartphones as the transceiver to identify target materials. The fundamental idea of our design is that acoustic signals, when propagated through different materials, reach the receiver via multiple paths, producing distinct multipath profiles. These profiles can serve as fingerprints for material identification. We captured and extracted them using acoustic signals, calculated channel impulse response (CIR) measurements, and then extracted image features from the time–frequency domain feature graphs, including histogram of oriented gradient (HOG) and gray-level co-occurrence matrix (GLCM) image features. Furthermore, we adopted the error-correcting output code (ECOC) learning method combined with the majority voting method to identify target materials. We built a prototype for this paper using three mobile phones based on the Android platform. The results from three different solid and liquid materials in varied multipath environments reveal that our design can achieve average identification accuracies of 90% and 97%.

## 1. Introduction

How many times have you taken a sip to clear a subway security check? Material identification is also a major application in warehouses and workshops, where it can help inspect and inventory materials to prevent accounting inaccuracies. Many industries are looking to solve these problems and improve productivity by deploying equipment and instruments [1,2]. For example, Agilent’s Chemical Analysis Group [1] leverages instruments to identify how much gold is in ore. Current instruments, however, can only identify targets by nebulizing the materials or inserting probes, which damage the target. In recent years, researchers have looked into non-contact material identification models.

The most popular non-contact material identification models are based on the following signals: radio frequency (RF) signals [3,4,5], ultra-wideband (UWB) signals [6,7,8], radar signals [9], and Wi-Fi signals [10]. For example, TagScan [5] uses RF signals to identify similar liquids, such as Pepsi and Coke. WiMate [10] can identify the board and paperboard using Wi-Fi signals. However, these models are not portable and generally rely on expensive equipment. Many applications, however, would benefit from knowing a target’s material. For example, in security, identifying liquid materials can determine whether a liquid carried by a passenger is prohibited during the security check, so as to ensure the safety of public transport. Similarly, in daily lives, it can help the visually impaired obtain information from the outside world, such as the liquids they wish to drink, and hard solids surrounding them.

This paper introduces an acoustic signal-based material identification model that functions in various environments with different degrees of multipath effects. Acoustic signals play a pivotal role in the current intelligent perception, finding extensive applications [11,12,13]. Unlike previous approaches to model construction [14,15,16,17,18], our design uses the built-in microphone and speaker of a mobile phone as the receiver (RX) and transmitter (TX), making it portable and commercially off-the-shelf (COTS). The underlying intuition of our design is that the multipath effects vary when acoustic signals propagate in different materials, forming the basis for the acoustic signal-based material identification that can be used for identifying different materials.

Specifically, acoustic signals reach the RX via different multiple paths, leading to different distortions, resulting in different channel impulse response (CIR) measurements when identified in different materials. However, the received CIR measurements differentiate between solid and liquid materials due to the unique physical properties of acoustic signal propagation in them [19]. So, how can we extract the same features to obtain the CIR changes of different solids and liquids? To do so, we need to extract image features based on time–frequency domain feature graphs. During material identification, the time-frequency domain features can first be extracted and graphed; we then extract the histogram of oriented gradient (HOG) and gray-level co-occurrence matrix (GLCM) image features as the characteristics used in material identification. Then, we leverage error-correcting output codes (ECOCs) to generate respective identification models, and then employ the majority voting method to obtain the identification results.

**Summary of results:** We built a prototype of our design using the speaker and microphone embedded in smart devices and evaluated it with three types of solids and liquids in three environments with different multipath effects. We designed multiple experiments to evaluate the effectiveness of our design, e.g., we compared the performance with and without image features or the majority voting methods; we compared the classification model of material identification with six commonly used classification models, etc. We also evaluated the robustness of the method in terms of different mobile phones, the surrounding environment, etc. In our solution, we found that the time–frequency domain feature graphs are more clearly distinguishable between the different materials than the features. In addition, the average accuracies of our design in identifying three different solid and liquid materials are approximately 90.45% and 96.72%, the empty cup identification average accuracy is higher than 99.21%, and our design has strong robustness.

In this paper, we make the following contributions:

1. We present a material identification model based on acoustic signals, which has high accuracy and strong robustness in various environments. At the same time, our design is a general identification model, which identifies solid and liquid materials as well as identifies whether it is an empty cup, without sacrificing accuracy.
2. We demonstrate that image features extracted from graphs based on time–frequency domain features are better applied to material identification than time–frequency domain features.
3. We present a method to improve the identification accuracy of the model using ECOC combined with the majority voting method. Although our design is based on acoustic signals, the basic method can be extended to other wireless signal-based models.

The remainder of this article is structured as follows. Section 2 provides a brief introduction to the principles and feasibility analysis of material identification using acoustic signals, as well as the principles of ECOC learning. In Section 3, the entire process of the proposed method is explained in detail. Section 4 presents the experimental setup details and comparative experiments to validate the effectiveness and robustness of the proposed method. Section 5 discusses the related work on current material identification. Section 6 discusses our design, while Section 7 presents a conclusion.

## 2. Preliminary

### 2.1. Acoustic Signals

**Figure 1:**
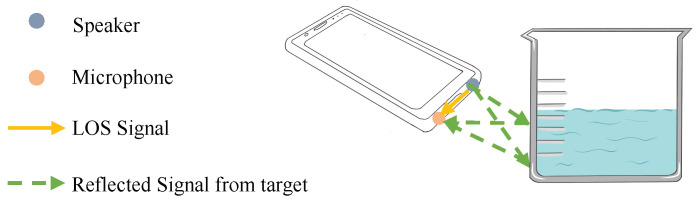
The rationale of acoustic signal-based material identification. Due to the multipath effect, the acoustic signals have different degrees of attenuation after being reflected by the target, which can be used as a fingerprint for material identification.

The basic principle of material identification based on acoustic signals consists of physical properties of signals as they propagate through materials. In actual scenarios, when high-frequency acoustic signals are emitted from the speaker, due to the existence of obstacles in the surrounding environment, the signals propagate through the target and arrive at the RX through reflection, refraction, and attenuation, which is called the multipath effect, as shown in Figure 1. When the signals propagate through different materials, the multipath effect will cause different distortions, which can be used as the fingerprints of material identification. Regarding multipath propagation, mobile devices (e.g., mobile phones) can estimate the CIR of the reflected signal frames, which feature the propagated signal paths [20].

Acoustic channels can be modeled as linear time-invariant systems, which effectively model signal attenuation and propagation delay on multiple propagation paths [21]. In a system with a microphone and speaker as the transceiver, let X(t), Y(t), and H(t) separately be the transmitted signal, the received signal, and the CIR of the acoustic signal measured [22]. The CIR measurements are obtained through inverse fast Fourier transform (IFFT); they describe how acoustic signals propagate from TX and RX and how the surrounding environments affect the acoustic signals. Therefore, the H(t) is as follows in Equation (1):

(1) $$H\left(t\right)=\frac{Y(t)}{X(t)}$$

In addition, it captures the multipath signals from the target and other surrounding environments, the target will, therefore, generate unique patterns in the measured CIR that can be leveraged for material identification.

In practice, the TX modulates and continuously sends inaudible high-frequency acoustic signals frame; the frame is then reflected from the target and received by the RX. The CIR measurements are expressed with a group of complex values, and the corresponding frequency and spectrogram of the CIR can be obtained.

### 2.2. How Different Materials Affect CIR

The acoustic signal-based material identification model relies on the repeatable collection and, therefore, on similar CIR measurements. In the identification system, mobile devices can be leveraged to identify the target materials. However, it is not clear how different materials will affect CIR, and how CIR measurements will reflect their characteristics. Through the above analysis, in the following paragraphs, we are going to deeply seek the connections between the material and the measured CIR.

**Figure 2:**
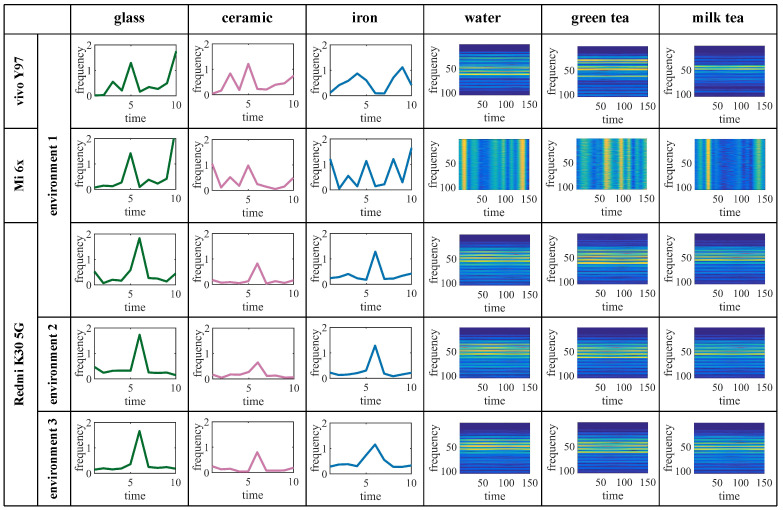
CIR frequency domain feature graphs of different materials.

**Different materials.** Some researchers have designed experiments and found that the wireless signal-based time–frequency domain features extracted, such as the phase [5], power spectral density (PSD) [23], etc., can be used to identify the target materials, which proves that the time–frequency domain features are different between different materials. We investigate images such as CIR frequency for different solids and short-time Fourier transform (STFT) for different liquids; the results are shown in Figure 2. It can be seen that there are significant differences in the frequency and STFT images for different solid and liquid materials, which are the CIR measurements as fingerprints for material identification.

**Different phones.** Our design relies on the microphone and speaker of the mobile phone as the transceiver. The advantage is that there is no need to configure redundant equipment, and it is convenient and easy to operate. The concern is that the location and model of speakers and microphones may vary between mobile phones, and the final CIR measurement can be different. In order to verify if the difference will affect the identification of materials, a comparative experiment was designed using three different mobile phones, including Redmi K30 5G, Mi 6x, and vivo Y97 to collect acoustic signals of the same material and draw CIR frequency domain feature graphs. It was found that there are differences in the frequency domain feature graphs of different materials under the same mobile phone, as shown in Figure 2. Experiments show that different mobile phones do not significantly impact material identification, ensuring that the model is generalizable among different mobile phones.

**Different environments.** In the process of collecting acoustic signals, the collection environment of the signals will always experience changes, sometimes with weaker and other times with stronger multipath environments, leading to different CIR measurements. To explore this issue, we artificially increased the number of facilities around the experiment to change the multipath environments. We then observed the differences in the CIR frequency domain graphs of the same material in different multipath environments, as shown in Figure 2. Experiments show that the graphs tend to be the same in different multipath environments, indicating that our design can identify materials regardless of the multipath effect of the environment.

### 2.3. ECOC

The steps involved in solving multiple binary classification problems based on ECOC are encoding, training, and decoding [24].

**Figure 3:**
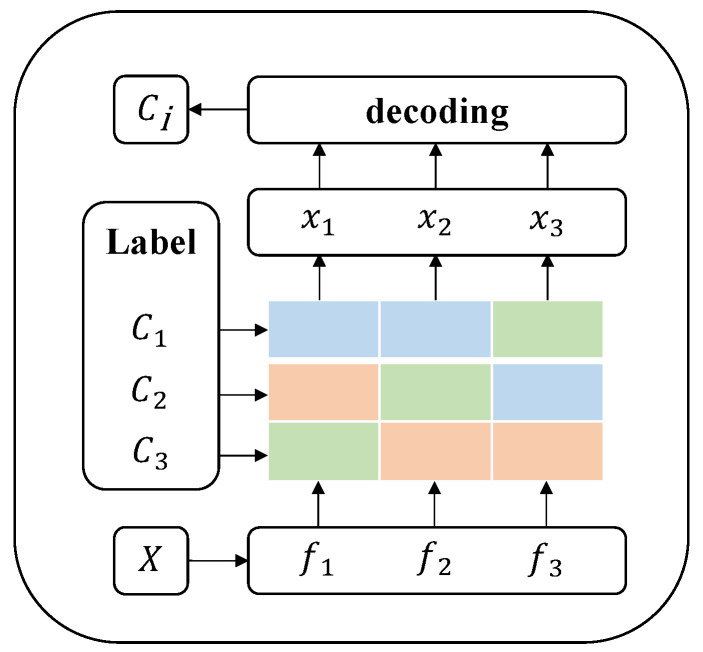
“One -versus-one” coding matrix.

In this paper, the predefined coding method is used, i.e., the coding method that does not depend on the sample and determines the coding matrix before classification. We use the “one-versus-one” coding matrix, as shown in Figure 3.

The colored part in the figure is the coding matrix. If *i* labels are given, the size of the coding matrix is i×i, the *i*th row in the coding matrix represents the code of the *i*th label $C_{i}\left(i=1,2,3\right)$, and the *i*th column represents a binary classification result. We use three different colors—green, blue, and pink—to represent code elements “0”, “1”, and “−1”, respectively, where “0” indicates that the label corresponding to the row does not participate in the training of the second classifier; “1” and “−1” represent the two labels that participate in this binary classifier training. In the training stage, a binary classifier is trained for every two labels in the *i* labels. Each binary classifier is called a base classifier $f_{j}$, and there are a total of $\frac{i(i-1)}{2}$ base classifiers. In the training stage, the training sample first divides the data into the required two data labels, according to column *j* in the coding array corresponding to *i*, and then trains the corresponding base classifiers “$f_{1}$, $f_{2}$, $f_{3}$”. In the test stage, we classify the test sample *X* through all base classifiers to obtain the code ($X_{1}$, $X_{2}$, $X_{3}$) of the sample (where $X_{n}\in \left\{-1,1\right\}$). Next, for each encoding vector $X_{n}$, the most similar encoding vector is searched for in the output results of all classifiers, resulting in a predicted category $c_{i}$. Typically, the Hamming distance is used to measure the similarity between two binary vectors.

The Hamming distance is a measure of the difference between two strings of equal lengths. In other words, it is the count of the number of positions where the *k*th character in one string is not equal to the *k*th character in the other string. The formula for the Hamming distance [25] is shown below in Equation (2):

(2) $$D\left(x,y\right)=\sum_{i=1}^{n}\left(x_{k}\neq y_{k}\right)$$

where *x* and *y* are two strings of equal lengths with *m* characters, and $x_{k}$ and $y_{k}$ are the *k* characters of the two strings, respectively.

## 3. Test Procedures

### 3.1. Overview

**Figure 4:**
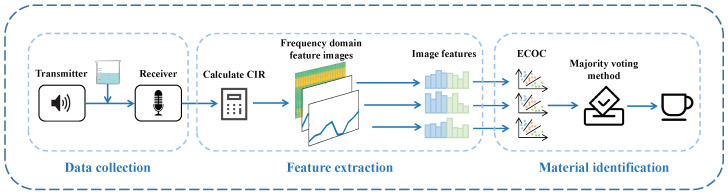
Overall material identification framework.

Figure 4 shows the overall identification framework of the acoustic signal-based material identification model. It mainly consists of three steps: data collection, feature extraction, and material identification. During the data collection step, the speaker emits inaudible acoustic signals, and the microphone receives the returned acoustic signals. In the feature extraction step, the CIR measurements of acoustic signals are calculated, the time–frequency domain features in the CIR are extracted and drawn into graphs, and then HOG and GLCM image features are extracted and combined for identification. In the material identification step, we use the ECOC learning method combined with the majority voting method to obtain the identification results. We will introduce the details of the model design in the next subsection.

### 3.2. Data Collection

We use the built-in microphone and speaker of the mobile phone as the RX and TX, respectively. The speaker transmits the acoustic signals, and the microphone collects the acoustic signals that propagate back through the target. We investigate three (solid and liquid) materials to demonstrate the feasibility of our design, and collect 45 groups of acoustic signals from different materials. When collecting acoustic signals, each group of signals is recorded for about 8 s. Acoustic signals are saved in WAV (waveform audio) files for later feature extraction. The WAV file is currently the industry standard for audio preservation because it can be edited and processed by most applications [26]. Since the acoustic signals are acquired in two channels, the signals are displayed as two columns of data.

### 3.3. Feature Extraction

In the feature extraction step, only the relatively stable part of the middle segment of the acoustic signals is used. We found that when drawing time–frequency domain features, such as frequency, short-term energy (STE), and Mel frequency cepstral coefficient (MFCC) into a graph, the differences between different materials are very obvious. Therefore, we use the image features extracted after drawing the time–frequency domain features into graphs as material identification and empty cup identification features.

**Time–frequency domain features.** We extract the raw acoustic signals and a variety of time–frequency domain features, which can be drawn into graphs; the feature descriptions are shown in Table 1.

**Image features based on the time–frequency domain feature graph.** We draw the above time–frequency domain features into color graphs, and extract image features based on graphs for material identification. Inspired by prior work [33,34,35,36], we calculated HOG and GLCM features.

**Figure 5:**
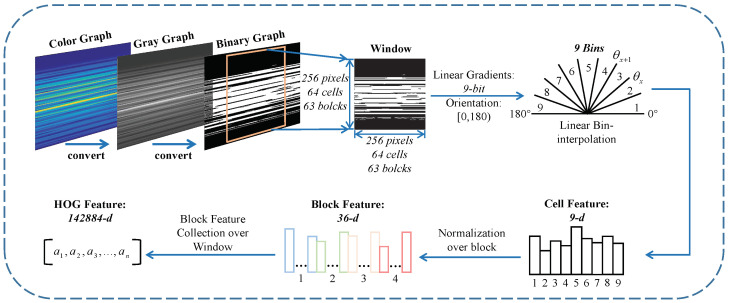
The HOG feature extraction pipeline.

For HOG features, the focus is to extract edge orientations and distribution. There is a HOG feature extraction pipeline, as shown in Figure 5. We convert the three-channel, 24-bit color graph to a one-channel gray graph, using a suitable threshold found by the Otsu method [37,38]; afterward, we use this threshold to convert to a binary graph for linear-gradient calculation. HOG features describe the geometric edge distribution within the graph window containing 256×256 pixels. The window is divided into non-overlapping 4×4-pixel patches, which are called cells. Moreover, a block consists of 2×2 cells and overlaps with adjacent blocks by one cell, resulting in 63×63 blocks within a window. The horizontal gradient $g_{x}$ [39] and vertical gradient $g_{y}$ [39] of pixel $p_{x,y}$ at row *x* and column *y* are expressed as Equations (3) and (4):

(3) $$g_{x}=p_{x+1,y}-p_{x-1,y}$$

(4) $$g_{y}=p_{x,y+1}-p_{x,y-1}$$

We calculate the gradient magnitude $g_{x,y}$ [39] and orientation θ [39] bin, which can be calculated according to Equations (5) and (6):

(5) $$g_{x,y}=\sqrt{g_{x}^{2}+g_{y}^{2}}$$

(6) $$\theta =\arctan\frac{g_{y}}{g_{x}}$$

As shown in Figure 5, the limit angles $\theta_{x}$ and $\theta_{x+1}$ of each bin are known constants. For each cell, the data are accumulated into nine bins of a local histogram and normalized. The 3969 (63×63 blocks) 36-*d* block features form a 142,884-*d* (3969×36) HOG feature, denoted as $HOG=[a_{1},a_{2},...,a_{n}]$.

**Figure 6:**
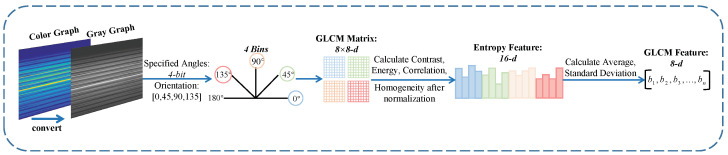
The GLCM feature extraction pipeline.

For GLCM features, the focus is on extracting contrast, homogeneity, correlation, and energy. Figure 6 shows our GLCM feature extraction pipeline. Similarly, we convert the color graph to a gray graph. Each matrix value of the GLCM describes the probability value of the change between gray levels *x* and *y* for a particular angle and displacement. For a given distance *d*, the GLCMs of four angles are defined for θ = 0°, 45°, 90°, and 135°, denoted as $g\left(d,0\right)$, $g\left(d,45\right)$, $g\left(d,90\right)$, $g\left(d,135\right)$.

Afterward, to prevent negative entropy values, we normalize the four GLCMs, calculate the contrast [40], energy [41], correlation [42], and homogeneity [41] to combine them into a 4×4 matrix, which can be expressed separately as Equations (7)–(10):

(7) $$Contrast=\sum_{x}\sum_{y}\left[{\left(x-y\right)}^{2}p\left(x,y\right)\right]$$

(8) $$Energy=\sum_{x}\sum_{y}p{\left(x,y\right)}^{2}$$

(9) $$Correlation=\frac{\left[\sum_{x}\sum_{y}\left(xy\right)p\left(x,y\right)-\alpha_{i}\alpha_{j}\right]}{\beta_{i}\beta_{j}}$$

(10) $$Homogeneity=\sum_{x}\sum_{y}\frac{p\left(x,y\right)}{1+{\left(x-y\right)}^{2}}$$

where $p\left(x,y\right)$ represents the frequency of each element, $\alpha_{i}$ and $\alpha_{j}$ denote the averages, while $\beta_{i}$, $\beta_{j}$ represents the mean square deviation.

Next, we calculate the average and standard deviation of each column of the matrix, which are combined to form an 8-*d* GLCM feature, denoted as $GLCM=[b_{1},b_{2},...,b_{m}]$.

Finally, we combine the HOG and GLCM features as material identification features, denoted by $Feature=[a_{1},a_{2},...,a_{n},b_{1},b_{2},...,b_{m}]$.

### 3.4. Material Identification

**Figure 7:**
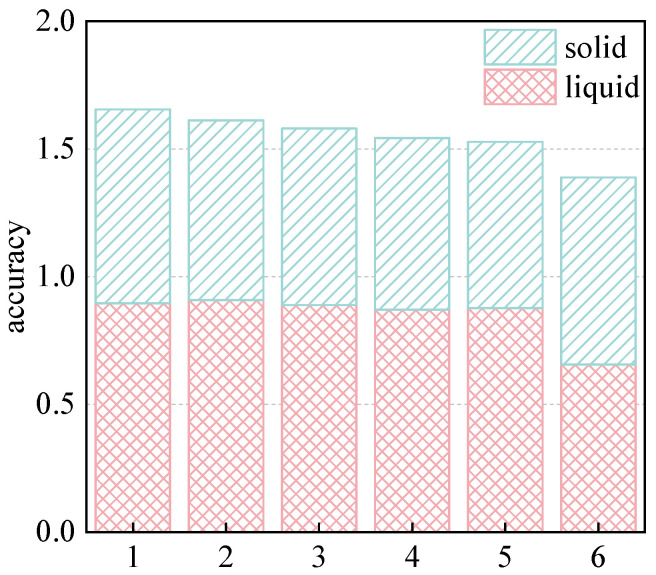
The histogram of material identification accuracy under different frequency domain feature graphs.

In order to decide which time–frequency domain feature graph combinations can perform better with the majority voting method, we draw the various time–frequency domain features extracted in the above subsection into graphs, extract the image features, and input them into the ECOC to obtain the average material identification accuracy, as shown in Figure 7 (only the features with both solid and liquid identification above 70% accuracies are shown, and the feature interpretations are shown in Table 2). Experimental results show that the identification accuracies of image features based on the frequency domain features are better than those based on time domain features.

In addition, we also calculate the mutual information between the above features and all labels, as shown in Table 3. Mutual information is a measure of the relationship between two variables. When calculating the mutual information between each feature and class, we use three types of entropy: the entropy of the current feature H(x) [43], the joint entropy of the feature given the class H(x,y) [43], and the entropy of the current class H(y) [43]. Shannon entropy is one of the most commonly used methods for calculating entropy in information theory. It is used to measure the purity or uncertainty of a dataset. In the field of machine learning, Shannon entropy is often used to calculate feature importance. By calculating mutual information [43] based on Shannon entropy, we can obtain the degree of correlation between features and perform reasonable feature selection. The formulas for the three types of entropy mentioned above are as follows in Equations (11)–(14):

(11) $$H\left(x\right)=-\sum P\left(x_{i}\right)logP\left(x_{i}\right)$$

(12) $$H\left(x,y\right)=-\sum P\left(x_{i},y_{j}\right)logP\left(x_{i},y_{j}\right)$$

(13) $$H\left(y\right)=\sum P\left(y_{j}\right)logP\left(y_{j}\right)$$

(14) Mutual_Information=H(x)+H(y)−H(x,y)

where *i* represents the sample number and *j* represents the class number.

Based on the observations from Table 3, we can conclude that the first three frequency domain feature graphs are more suitable for material identification tasks.

Finally, we select the first three frequency domain feature graphs as the features to identify the target materials using the majority voting method. The remaining extracted features can also be used in combination with the majority voting method, which may improve the model’s identification accuracy to some extent.

We use the ECOC [44] learning method for material identification, which consists of multiple binary classification algorithms of support vector machine (SVM). This method can convert multi-classification problems into multiple binary classification problems for resolution. The solution steps include encoding, training, and decoding. In our design, the “one-versus-one” coding matrix is used, which is the predefined coding method. In the training stage, a binary classifier is trained for every two labels in the *i* labels, which is called a base classifier. Afterward, we classify the test data through all base classifiers to obtain the data code and finally decode it to retrieve the predicted label.

To maximize the advantage of selected frequency domain feature graphs, we construct a separate classifier for each graph after extracting image features. We built three classifiers using the ECOC learning method, inputting image features to all classifiers, and obtaining a decision from each one. Each ECOC classifier uses a one-versus-one encoding matrix to encode and decode image features to obtain majority class labels. The final result is calculated by using the majority voting method for all ECOC classifier decisions.

## 4. Test Apparatus and Results

### 4.1. Test Apparatus

**Figure 8:**
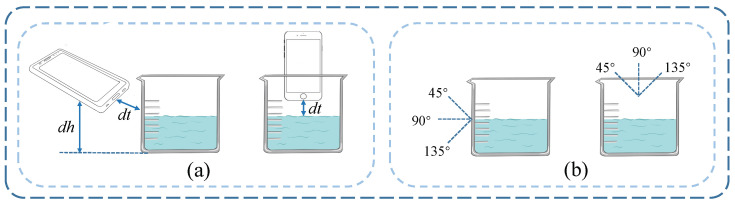
Device deployment; (**a**) the device deployment for different distances of the transceiver, (**b**) the device deployment for different angles of the transceiver.

**Device deployment.** There are two types of equipment deployment, as shown in Figure 8a. The first is that the transceiver is parallel to the horizontal, the transceiver is kept at a distance of 1 cm (dt) from the measured target, and a height of 3 cm (dh) from the target placement plane. The second is that the transceiver is perpendicular to the horizontal. At this time, the transceiver is perpendicular to the target and needs to maintain a distance of 1 cm from it. In the data collection stage, the target is placed on a flat surface, and the position of the target, the position of the transceiver, and the surrounding environment are fixed.

**Figure 9:**
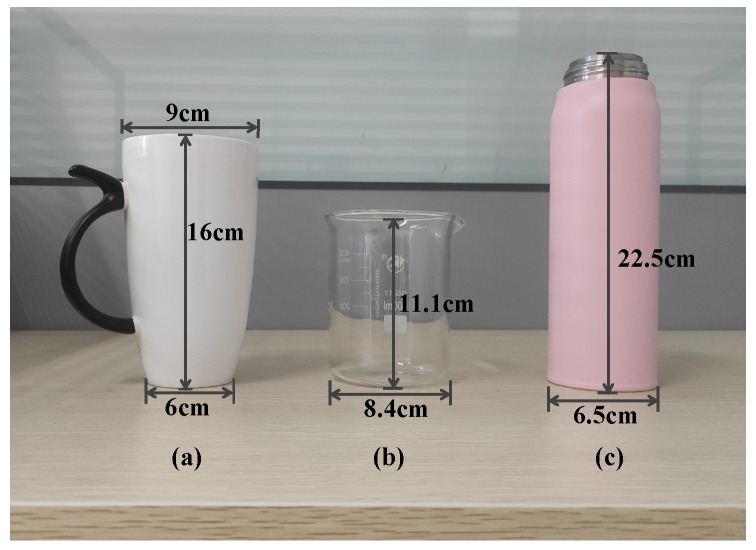
The shapes and dimensions of three solid targets; (**a**) ceramic cup, (**b**) glass pup, (**c**) stainless steel cup.

**Experimental setup.** We evaluate our system using three different solid (ceramic, glass, stainless steel) and liquid materials (water, milk tea, and green tea). The shapes and dimensions of the three solid targets are shown in Figure 9. To collect data for the liquid target, we introduced 200 mL of liquid into the identical container (Figure 9b) with a container thickness of 2 mm. To comprehensively verify the feasibility of the system, we designed three types of experiments, including solid and liquid material identification and empty cup identification. Furthermore, three angles of 45°, 90°, and 135° were designed in the experiment to evaluate the influence of the transceiver angle on the material identification, as shown in Figure 8b. We also set up experiments to change the distance dt, including 1 cm, 2 cm, 3 cm, 4 cm, and 5 cm, to verify the robustness of our design. Moreover, to research the influence of different multipath environments, we designed three levels of multipath environments, namely a weak multipath environment, a medium multipath environment, and a strong multipath environment, artificially increasing the number of objects in the same environment to modify the multipath effects. These environments are labeled as environment 1, environment 2, and environment 3, respectively. In the weak multipath environment, the signals encounter small time delays and relatively clear propagation paths. Only some weak attenuation or reflection paths may exist. In the medium multipath environment, the number of multipath components in the signal increases. In the strong multipath environment, the transmission quality of the signal is affected, and it may experience interference.

**Parameter settings.** The sampling rate of the acoustic signals emitted by the TX is set to 48 KHz because the sampling rate of built-in microphones and speakers in current COTS mobile phones is usually 48 kHz [12]. Therefore, the acoustic signal sampling rate used in this model is suitable for most mobile phones. Moreover, the frequency of the transmitted signals is between 18 and 22 kHz [45]. Due to the acoustic signals passing through processes such as reflection, refraction, and diffraction from the transmitter, the signals received at the receiver are a mixture of the target signals and background noise. The frequency is relatively complex, and the waveform contains many sharp spikes and irregular amplitudes.

**Method evaluation.** In order to describe the performance of the model, we also consider evaluation metrics, such as accuracy, precision, F-score, etc. Precision refers to the proportion of all true positive classes that are judged as positive classes. The F-score combines the results of precision and recall.

**Model evaluation.** We compared ECOC with five commonly used machine learning methods, including the K-means clustering algorithm (K-means), SVM, random forest (RF), naive Bayes (NB), and K-nearest neighbor (KNN). We used the linear kernel and RBF kernel as kernel functions for SVM. In addition, since there may exist both linear and non-linear relationships among features, we also compare the performance of a simple neural network (backpropagation neural network, BP). In the BP neural network, the loss function choice has a significant impact on training model performance, as it is used to measure the difference between network outputs and true labels. Therefore, choosing an appropriate loss function can better guide the network’s learning process and improve the model’s effectiveness and accuracy. Here, we employ the commonly used cross-entropy H(p,q) [46] and Kullback–Leibler divergence (KL divergence) $D_{K}L\left(p||q\right)$ [47] as the loss functions for the BP neural network, with Equations (15) and (16):

(15) $$H\left(p,q\right)=-\sum_{i=1}^{n}p_{i}log\left(q_{i}\right)$$

(16) $$D_{K}L\left(p||q\right)=\sum_{i=1}^{n}p_{i}log\left(\frac{p_{i}}{q_{i}}\right)$$

where *p* represents the true probability distribution and *q* represents the predicted probability distribution.

### 4.2. Overall System Performance

**Figure 10:**
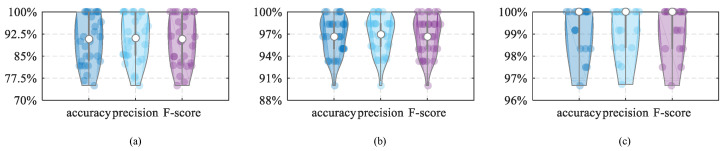
Overall performance; (**a**) solid material identification performance, (**b**) liquid material identification performance, (**c**) empty cup identification performance.

We average the material identification and empty cup identification results under different mobile phones to obtain the final overall performance; the main results of the evaluation are as follows:

1. The solid and liquid material identification performances are shown in Figure 10. The results show that the model accuracies are about 90.4% and 96.7%, respectively. Moreover, the average precision and F1-score of solid material identification are 91.1% and 90.3%, while the average precision and F1-score for liquid material identification are 96.9% and 96.7%. Moreover, the empty cup identification performance is also shown in Figure 10. The accuracy, precision, and F-score are higher than 99.2%.
2. Compared with other material identification models, our design has stronger robustness, which is not affected by changes in light and the surrounding environment. Moreover, it only needs a speaker and microphone that can transmit and receive acoustic signals during material and empty cup identification, which are now available in smartphones. The above shows that our design is convenient.

In all experiments, we use two-fold cross-validation to evaluate the stability of the model and run it multiple times to calculate the average as the final identification result. In order to ensure that the training data and test data do not overlap, the dataset is randomly divided into two equal parts.

Our results show that the model can identify the material of the solid and liquid with an accuracy of over 90.45% and 96.72%, and can identify the empty cup with an accuracy of about 99.21%.

It can be found from Figure 10 that this model has a higher accuracy of liquid material identification than solid material, which may be caused by the following two situations. The first possible situation is that the acoustic signals are reflected, refracted, and attenuated differently in solids and liquids [19], resulting in different identification results. Moreover, the extracted features are more suitable for liquid materials, which may lead to lower identification results for solid materials.

### 4.3. Evaluation of Effectiveness

To demonstrate the effectiveness of this model, in the experiment, we evaluate the impact on the performance with and without the majority voting method and image features, and then compare the identification model used in our design with the currently used models, taking the vivo Y97 mobile phone as an example. Moreover, the experimental setup involves data collection in a weak multipath environment, with the transceiver placed parallel to the target surface at a height of 3 cm and maintained at a distance of 1 cm from the target.

#### 4.3.1. Feature Extraction Analysis

We compare our feature extraction method from the two following aspects: (1) the effect of the image feature on performance and (2) the effect of the majority voting method on performance.

**Impact of the extracted image features.**

*Our results show that extracting image features (after drawing the frequency domain features into a graph) results in a higher identification performance than extracting frequency domain features to identify solid and liquid materials.*

**Figure 11:**
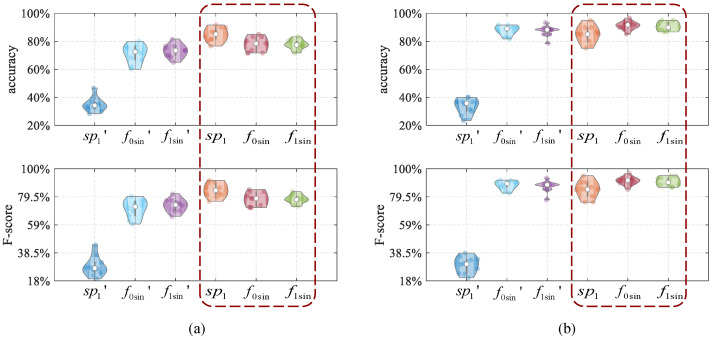
The comparison results of accuracy and F-score in material identification performances of extracted and non-extracted image features; (**a**) solid material identification performance, (**b**) liquid material identification performance.

To verify the influence of image features on the identification performance, we perform material identification based on the above frequency domain features and the image features extracted after being drawn into graphs, and compare the performances of different features, as shown in Figure 11. ${sp_{1}}^{\prime }$, ${f_{0sin}}^{\prime }$, ${f_{1sin}}^{\prime }$ are the raw frequency domain features. The same training dataset and test dataset are used in the experiment, and the specific performances are shown in Table 4. Experiments show that image features based on frequency domain feature graphs are for material identification rather than frequency domain features.

From Table 4, we can see that the accuracy of material identification using spectrogram features is 34%, and the accuracy using spectrogram image features is about 84%. It can clearly be seen that the identification accuracy after extracting image features is better because the spectrogram is a color graph, which will contain more useful information than the data itself. The frequency features, such as $f_{0sin}$ and $f_{1sin}$, as well as the extraction and non-extraction of image features, are not too different, but the accuracy is still improved by about 5%.

**Impact of the majority voting method.**

*Our results show that the majority voting method for solid and liquid material identification can improve identification performance.*

**Figure 12:**
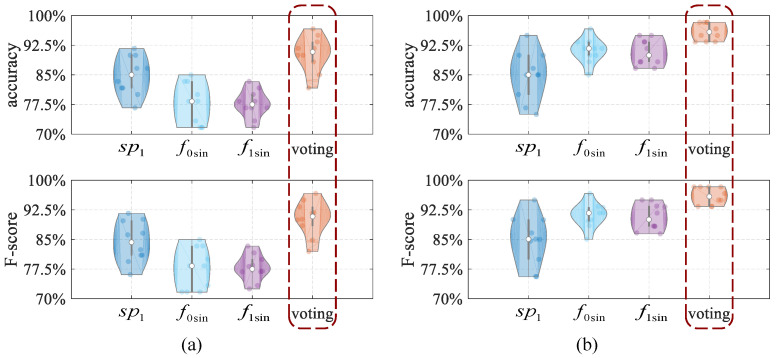
The comparison results of accuracy and F-score in material identification performance with and without the majority voting method; (**a**) solid material identification performance, (**b**) liquid material identification performance.

In order to verify the influence of the majority voting method on the identification performance, we extract the image features from three frequency domain feature graphs and input them into ECOC, respectively, to obtain the identification accuracies. Then, we combine them with the majority voting method to obtain the identification accuracy and compare the performances, as shown in Figure 12. In the experiment, the training dataset and test dataset are the same, and the identification accuracy, precision, and F-score are shown in Table 5. Experiments show that the majority voting method can effectively improve identification performance.

For solid material identification, the identification accuracy of the majority voting method is about 90%, and the identification accuracy using an individual graph is obviously lower. Among them, the lowest accuracy is about 10% different from the accuracy of the majority voting method, and the highest accuracy is also 6% lower than it. For liquid material identification, the identification accuracy of the majority voting method is more than 95%, while in the identification accuracy using an individual graph, the highest accuracy does not exceed 92%, and the lowest accuracy is about 85%. The majority voting method not only works well for solid identification, with an accuracy of about 90%, but also for liquid identification, with an accuracy of about 96%.

#### 4.3.2. Classification Model Analysis

We also compare our ECOC-based classification model against the currently used machine learning methods.

*The results show that compared with other learning methods, ECOC can be well-applied to material identification with obvious performance improvement.*

**Figure 13:**
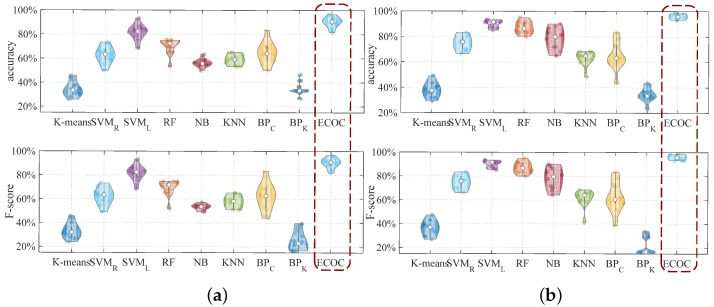
The comparison results of the accuracy and F-score of material identification performance using different classification models; (**a**) solid material identification performance, (**b**) liquid material identification performance.

We compare ECOC with five commonly used machine learning methods, including the K-means clustering algorithm (K-means), SVM, random forest (RF), naive Bayes (NB), and K-nearest neighbor (KNN). We use the linear kernel and RBF kernel as kernel functions for SVM. In addition, since both linear and non-linear relationships may exist among features, we also look at the performance of a simple neural network (backpropagation neural network, BP). In the BP neural network, the choice of loss function has a significant impact on training model performance, as it is used to measure the difference between network outputs and true labels. Therefore, choosing an appropriate loss function can better guide the network learning process, as well as improve model effectiveness and accuracy. Here, we employ the commonly used cross-entropy H(p,q) and Kullback–Leibler divergence (KL divergence) $D_{K}L\left(p||q\right)$ as the loss functions for the BP neural network, with the following formulas:

To evaluate the performance of ECOC, we compare ECOC with eight commonly used classification methods, including the K-means, SVM with RBF kernel, SVM with linear kernel, RF, NB, KNN, BP neural network (cross-entropy), and the BP neural network (KL divergence). In the experiments, the extracted features and training and testing datasets are the same; all classification models are combined with the majority voting method. The results are shown in Table 6. Figure 13 shows the comparison results of the above classification models. Experimental results show that ECOC has the best material identification performance compared to other classification models.

K-means only divides the data into multiple clusters, and each cluster has no definite label, so we set an appropriate label for each cluster through multiple tests to obtain the highest accuracy. We also set the K value for KNN to make the best identification performance. For solid and liquid material identification, compared with ECOC, the other eight models perform poorly; the SVM with linear kernel has better identification accuracy but is still about average (7% lower than ECOC); and K-means has the worst performance, which is about 50% lower than ECOC.

### 4.4. Evaluation of Robustness

In order to demonstrate the robustness of our design, experiments are designed to compare material identification performance effects of different mobile phones, the surrounding environment, different angles to the transceiver, and different distances between the transceiver and the target. The experimental results show that none of the above has a significant impact on the performance of our design. In addition to the comparison of different mobile phones, the phones for collecting acoustic signals of solid and liquid materials are Mi 6x and vivo Y97, respectively.

#### 4.4.1. Impacts of Different Mobile Phones

*Our results show that different mobile phones perform well for solid and liquid material identification.*

In order to study the influence of mobile phones on the performance of our design, experiments were designed using three different mobile phones to collect acoustic signals in the same environment (weak multipath environment) and device deployment (the transceiver was placed parallel to the target surface at a height of 3 cm and maintained at a distance of 1 cm from the target); the performance of material identification was then evaluated. The results are shown in Table 7. The experimental results show that our design can achieve high performance on the tested mobile phones.

For solid material identification, the identification accuracies in different mobile phones are all above 80%, and the highest identification accuracy reaches 99%. For liquid material identification, the identification accuracies of different mobile phones exceed 95%, and the highest accuracy is about 99%. It can be seen from Table 7 that the identification accuracy of Redmi K30 5G has the lowest performance, and the phone with the highest performance is Mi 6x, indicating that the model and location of the mobile phone’s microphone and speaker have an impact on our design, but the experimental performance obtained is acceptable.

#### 4.4.2. Impact of the Surrounding Environment

*The results show that the surrounding environment has little impact on the performance of this model.*

In order to study the influence of the surrounding environment on the performance of this model, we collect acoustic signals in three environments with different multipath conditions for material identification, as shown in Figure 8. During the data collection stage, the transceiver is positioned parallel to the target surface and maintained at a height of 3 cm, while ensuring a distance of 1 cm from the target. The results are shown in Table 8. Experiments show that the identification performance of this model remains stable in different multipath environments.

From Table 8, we can see that the accuracies of solid and liquid material identification can reach more than 99% and 94% in different multipath environments. The accuracy difference between different environments is less than 1% for solid material identification, and the difference between the highest and lowest accuracies in different environments does not exceed 2% for liquid material identification.

#### 4.4.3. Impacts of Different Angles on the Transceiver

*The experimental results show that the identification accuracies of solid and liquid materials can reach about 99% and 88% under different angles of the transceiver.*

In this experiment, material identification is trained and tested for different transceiver angles, as shown in Figure 8. The experimental conditions are ensured to be in a weak multipath environment, with the transceiver positioned parallel to the target surface at a height of 3 cm while maintaining a distance of 1 cm from the target. The performances of different transceiver angles are shown in Table 9. The experimental results show that our design can accurately accomplish material identification under different transceiver angles.

When the transceiver angle is 45°, the solid and liquid material identification accuracies are 99% and 88%. The identification accuracies of solid and liquid materials are about 99% and 96% when the angles are 90° and 135°. It can be seen that the variation in identification performance is minimal when the transceivers are set at different angles.

#### 4.4.4. Impacts of Different Distances on the Transceiver

*The experimental results show that the distance between the transceiver and the target does not have a large impact on material identification accuracy in this method.*

To evaluate the influence of the dt distance between the transceiver and the target on our model, five distances from 1 cm to 5 cm are designed in the experiment, as shown in Figure 8. The identification performance is shown in Table 10. In this case, the collection environment is characterized as a weak multipath environment, with the transceiver maintaining 90° with the target. We find that material identification performances at different distances do not change significantly.

We can see from Table 10 that for the solid material identification, the highest and lowest accuracies are 99.83%, 99.00%, and the difference between the highest and lowest accuracies is about 1%, which means that in the actual identification, different distances have no visible performance impacts on solid material identification. For the liquid material identification, the highest and lowest accuracies are about 96% and 90%, and the accuracies at different distances are all above 89%, which proves that in practical applications, liquid material identification at different distances performs well.

## 5. Material Identification-Related Work

The material identification method designed in this paper offers a comprehensive solution for identifying various materials, including solids and liquids, and in identifying the presence of liquids within containers. Our design primarily addresses issues related to factory security checks, waste sorting, and inventory management. Traditional material identification techniques can be broadly classified into two categories: contact-based and non-contact-based methods. However, both approaches have inherent advantages and limitations, as outlined in Table 11. To overcome these constraints, our method leverages acoustic signals, eliminating the need for physical contact that could potentially damage the materials under examination. Moreover, the utilization of common acoustic signals in our method ensures practical applicability in everyday scenarios. The portability and commercial availability of the transceiver devices further enhance the feasibility and accessibility of our design.

**Contact material identification method.** This method utilizes the differences in physical and chemical properties of different materials, applies instruments and equipment to qualitatively or quantitatively analyze them, and then identifies the material of the target [48,49]. For example, Techtron et al. [1] used atomic absorption spectroscopy for material identification. The method requires atomizing the sample into gas atoms, followed by a spectrometer, to form the light path. By analyzing the amount of light absorbed or emitted by the sample, the components of the sample and the content of each component are determined. However, using this method for material identification means that the sample used for identification will be completely damaged, and the cost is huge, which is not suitable for long-term use. In addition, permittivity is the resistance generated when the electric field is formed inside the liquid, so the permittivities of different liquids are basically different. Agilent Technologies [2] uses this knowledge to design instruments that realize the material identification of liquids. The instrument inserts the probe into the liquid and identifies liquids of different materials through the measured permittivity, which means that the probe needs to be checked and maintained frequently, and the instrument cannot complete the material identification for corrosive and conductive liquids.

**Non-contact material identification method.** At present, there are several popular methods for non-contact material identification: based on RF signals [3,4,5], based on UWB signals [6,7,8], based on radar signals [9], and based on Wi-Fi signals [10]. For example, TagScan [5] identifies the liquid by measuring the phase and the received signal intensity change when the RF signals penetrate through the liquid. The disadvantage is that RF signals are not widely popularized in daily life, and it is necessary to label each target in the early stage of material identification, which is a waste of resources. LiquID [6] uses UWB signals to estimate the permittivity of liquids to identify liquids. However, LiquID needs to use a specific container when identifying liquids, and the UWB system occupies a high bandwidth, which will interfere with other wireless communication systems during the operation. RadarCat [9] uses the random forest learning method to classify materials and objects based on radar signals, which can identify objects in real time. However, radar equipment is relatively expensive, and the radar signals are affected by background noise due to the high sensitivity of radar used by RadarCat. WiMate [10] realizes material identification according to the influence of objects on the amplitude and phase of Wi-Fi signals, and the identification performance is as high as 96.2%. However, Wi-Fi signals are susceptible to environmental interference.

## 6. Discussion and Limitations

In terms of improving the effectiveness and robustness of the identification model, there is room for further improvement in the performance of our design, which we will discuss below.

**Feasibility.** The material identification model based on acoustic signals in this paper is very portable and does not require a fixed transceiver. In actual scenarios, our design can help individuals who are blind when they drink water, as well as help individuals who are blind cook, by finding the condiments that they need. In addition, our design can also complete engineering material identification technology, which can improve the efficiency of factories. For the identification model used in our design, in addition to the above-mentioned six materials, this model can also identify a wider variety of materials as long as we add other kinds of materials to the training dataset.

**Surrounding environment.** This material identification model assumes that there is no human activity in the scene, which is the assumption of most of the current material identification models. When a user walks, the acoustic signals received by the RX are composite values of user activity and material information, and the received acoustic signals are difficult to separate. However, we believe that by combining the work by Luca Remaggi [50] and Masahiro Fukui [51], this problem can be solved, and it is also what we need to solve in future work to improve the robustness of the system.

**Cross-scene identification.** The identification model currently only tests the material’s identification accuracy in the same scene, i.e., the training data and the test data come from the same scene. However, in practical applications, material identification is performed via a cross-scene, and data collection is required for each material identification, which is time-consuming, labor-intensive, and impractical. Therefore, we can use the data augmentation technique designed by Wang et al. [21] to solve the problem of cross-scene identification. We only need to collect small amounts of raw data upfront, and then apply the technology to enhance the data. At this time, since the enhancement technology observes data in different scenarios, the data obtained by this technology can simulate different usage scenarios.

**Data collection.** In the early stage of this model, a large amount of training data need to be collected manually, which is time-consuming and cumbersome, and some scenarios may not be able to collect training data for a long time. At this point, we can solve this problem by splitting the data with the sliding window. In the early stage, the collection time of each data piece is about eight seconds, and in actual material identification, we do not need to use such a long data time to extract the required features. We only use a data time of 0.21 s in the experiment. Therefore, we can use the sliding window to find an optimal time length, and then divide the training data into multiple parts, each of which can be used for material identification. This solves the problem of manually collecting a large amount of data caused by the lack of training data.

## 7. Conclusions

In this paper, we propose a non-contact material identification model based on acoustic signals and frequency-graph features. The model is capable of distinguishing between different solid and liquid materials and can accurately identify—with high accuracy and strong robustness—whether a cup is empty. Our design demonstrates its feasibility by examining how materials affect the measurements of CIR. We extract image features using frequency domain feature graphs and utilize the ECOC learning method and majority voting method to construct the material identification model. Furthermore, we evaluate the performance of our design in terms of effectiveness and robustness. For effectiveness, we assess the use of majority voting methods and classifier selection and compare our method with commonly used features for material identification. Regarding robustness, we evaluate the accuracy of the model across different mobile phones and surrounding environments. The experimental results demonstrate the effectiveness and robustness of our model. The identification accuracies for solids and liquids reach 90% and 97%, respectively, while the accuracy for identifying empty cups reaches 99%. The impacts of different mobile phones, multipath environments, and transceiver settings on material identification performance are minimal. In addition, we believe that by combining our design with sliding window techniques, data augmentation, and signal separation technology, its practicality can be further enhanced. Additionally, incorporating additional features and classification models during the voting stage can lead to improved performance. However, these aspects are beyond the scope of this paper and will be explored in future work.

## Figures and Tables

**Table 1:** Time–frequency domain feature.

Label	Interpretation	Equation
**frequency**	apply the fast Fourier transform (FFT) to obtain the frequency.	/
**spectrogram [27]**	Amplitude graph of STFT of acoustic signals.	$spectrogram\left(m,f\right)={\left|STFT(m,f)\right|}^{2}$
**STFT [28]**	Fast Fourier Transform (FFT) the signals to obtain the STFT.	$STFT\left(m,f\right)=\sum_{n=-\infty }^{\infty }x\left(n\right)w\left(m-n\right)e^{-j2\pi fn}$
**raw signals**	Raw unprocessed acoustic signals.	/
**phase [29]**	calculated by determining the complex value’s angle.	$phase\left(n\right)=arg\left\{x\left(n\right)+j\hat{x}\left(n\right)\right\}$
**STE [30]**	Sum the squares of each frame signal to obtain the short-term energy.	$STE\left(n\right)=\sum_{i=n}^{n+N-1}x_{}^{2}\left(n\right)$
**envelope [31]**	Hilbert transforms the signals to obtain the envelope.	$envelope\left(n\right)=\sqrt{(x{\left(n\right)}^{2}+\hat{x}{\left(n\right)}^{2}}$
**MFCC [32]**	Calculate the MFCC using the relationship with frequency.	$f_{mel}\left(f\right)=2595\times \log_{}\left(1+\frac{f}{700\;\mathrm{Hz}}\right)$

**Table 2:** Frequency domain feature interpretations.

ID	Interpretation
**1/$f_{0sin}$**	frequency feature graph; all acoustic signal data; sine transformation the signals
**2/$sp_{1}$**	spectrogram graph; first column of acoustic signals data
**3/$f_{1sin}$**	frequency feature graph; first column of acoustic signal data; sine transformation the signals
**4/$f_{0cos}$**	frequency feature graph; all acoustic signal data; cosine transformation the signals
**5/$f_{1cos}$**	frequency feature graph; first column of acoustic signal data; cosine transformation the signals
**6/$f_{2sin}$**	frequency feature graph; second column of acoustic signal data; sine transformation the signals

**Table 3:** Mutual Information of different features.

	1/$f_{0\sin}$	2/${\mathrm{sp}}_{1}$	3/$f_{1\sin}$	4/$f_{0\cos}$	5/$f_{1\cos}$	6/$f_{2\sin}$
**Mutual Information**	**4.8299**	**4.8279**	**4.8003**	4.5079	3.6464	3.4329

**Table 4:** Identification performances of extracted and non-extracted image features.

	Solid Material Identification			Liquid Material Identification		
**Features**	**Accuracy**	**Precision**	**F-Score**	**Accuracy**	**Precision**	**F-Score**
${sp_{1}}^{\prime }$	35.00%	26.64%	27.86%	33.67%	38.62%	29.60%
${f_{0sin}}^{\prime }$	71.00%	73.75%	70.95%	88.00%	88.93%	88.08%
${f_{1sin}}^{\prime }$	73.00%	74.37%	73.15%	87.33%	88.40%	87.26%
** $sp_{1}$ **	**84.83%**	**85.32%**	**84.42%**	**84.83%**	**85.88%**	**84.76%**
** $f_{0sin}$ **	**77.67%**	**79.32%**	**77.67%**	**91.17%**	**91.67%**	**91.13%**
** $f_{1sin}$ **	**77.67%**	**79.68%**	**77.79%**	**90.67%**	**91.19%**	**90.65%**

**Table 5:** Identification performance with and without the majority voting method.

	Solid Material Identification			Liquid Material Identification		
**Method**	**Accuracy**	**Precision**	**F-Score**	**Accuracy**	**Precision**	**F-Score**
$sp_{1}$	84.83%	85.32%	84.42%	84.83%	85.88%	84.76%
$f_{0sin}$	77.67%	79.32%	77.67%	91.17%	91.67%	91.13%
$f_{1sin}$	77.67%	79.68%	77.79%	90.67%	91.19%	90.65%
**voting**	**90.33%**	**91.18%**	**90.36%**	**96.00%**	**96.08%**	**95.99%**

**Table 6:** Identification performances of different classification models.

	Solid Material Identification			Liquid Material Identification		
**Classification Models**	**Accuracy**	**Precision**	**F-Score**	**Accuracy**	**Precision**	**F-Score**
K-means	34.42%	34.47%	32.79%	37.67%	38.10%	36.08%
SVM with RBF kernel	62.67%	64.16%	62.15%	74.83%	77.38%	87.10%
SVM with linear kernel	81.67%	82.85%	81.70%	90.00%	90.64%	90.03%
RF	69.83%	70.39%	69.35%	87.17%	87.87%	87.10%
NB	56.17%	58.84%	53.33%	78.50%	81.67%	78.17%
KNN (K = 8)	58.83%	60.09%	57.77%	63.33%	66.63%	61.22%
BP (cross-entropy)	64.67%	75.45%	62.19%	35%	21.11%	24.48%
BP (KL Divergence)	64.83%	68.74%	62.40%	34.5%	17.25%	22.12%
**ECOC**	**90.33%**	**91.18%**	**90.36%**	**96.00%**	**96.08%**	**95.99%**

**Table 7:** Identification performances of different mobile phones.

	Solid Material Identification			Liquid Material Identification		
**Phone**	**Accuracy**	**Precision**	**F-Score**	**Accuracy**	**Precision**	**F-Score**
Redmi K30 5G	81.17%	82.33%	80.97%	95.17%	95.43%	95.16%
Mi 6x	99.83%	99.84%	99.83%	99.00%	99.08%	98.99%
vivo Y97	90.33%	91.18%	90.36%	96.00%	96.08%	95.99%

**Table 8:** Identification performance of the surrounding environment.

	Solid Material Identification			Liquid Material Identification		
**Environment**	**Accuracy**	**Precision**	**F-Score**	**Accuracy**	**Precision**	**F-Score**
environment 1	99.83%	99.84%	99.83%	96.00%	96.08%	95.99%
environment 2	99.83%	99.84%	99.83%	96.33%	96.62%	96.28%
environment 3	98.33%	98.41%	98.33%	94.83%	94.87%	94.83%

**Table 9:** Identification performances of different angles on the transceiver.

	Solid Material Identification			Liquid Material Identification		
**Angle**	**Accuracy**	**Precision**	**F-Score**	**Accuracy**	**Precision**	**F-Score**
45°	99.17%	99.22%	99.17%	88.5%	89.45%	88.68%
90°	99.83%	99.84%	99.83%	96.00%	96.08%	95.99%
135°	99.83%	99.84%	99.83%	96.00%	96.08%	95.99%

**Table 10:** Identification performances of different distances on the transceiver.

	Solid Material Identification			Liquid Material Identification		
**Distance**	**Accuracy**	**Precision**	**F-Score**	**Accuracy**	**Precision**	**F-Score**
1 cm	99.83%	99.84%	99.83%	96.00%	96.08%	95.99%
2 cm	99.58%	99.60%	99.58%	95.33%	95.69%	95.35%
3 cm	99.17%	99.21%	99.17%	89.33%	89.73%	89.25%
4 cm	99.00%	99.03%	98.99%	96.33%	96.46%	96.33%
5 cm	99.67%	99.68%	99.67%	96.00%	96.12%	95.99%

**Table 11:** Material identification-related work.

Models		References	Pros	Cons
**Contact material** **identification**	Equipment	Techtron-2013 [1]	high accuracy	material damage
		Agilent-2006 [2]		equipment maintenance
**Non-contact** **material** **identification**	RF	TagSca [5]	estimate the horizontal cut images of target	waste resources not universal signals
	UWB	LiquID [6]	lightweight	interfere with communication
	radar	RadarCat [9]	real-time identification	affected by noise high cost
	Wi-Fi	WiMate [10]	low-cost location independent	environmental interference

## Data Availability

The data presented in this study are available upon request from the corresponding author.
